# Parametric models to relate spike train and LFP dynamics with neural information processing

**DOI:** 10.3389/fncom.2012.00051

**Published:** 2012-07-24

**Authors:** Arpan Banerjee, Heather L. Dean, Bijan Pesaran

**Affiliations:** Center for Neural Science, New York UniversityNew York, NY, USA

**Keywords:** spike, LFP, information processing, rate coding, timing, decoding

## Abstract

Spike trains and local field potentials (LFPs) resulting from extracellular current flows provide a substrate for neural information processing. Understanding the neural code from simultaneous spike-field recordings and subsequent decoding of information processing events will have widespread applications. One way to demonstrate an understanding of the neural code, with particular advantages for the development of applications, is to formulate a parametric statistical model of neural activity and its covariates. Here, we propose a set of parametric spike-field models (unified models) that can be used with existing decoding algorithms to reveal the timing of task or stimulus specific processing. Our proposed unified modeling framework captures the effects of two important features of information processing: time-varying stimulus-driven inputs and ongoing background activity that occurs even in the absence of environmental inputs. We have applied this framework for decoding neural latencies in simulated and experimentally recorded spike-field sessions obtained from the lateral intraparietal area (LIP) of awake, behaving monkeys performing cued look-and-reach movements to spatial targets. Using both simulated and experimental data, we find that estimates of trial-by-trial parameters are not significantly affected by the presence of ongoing background activity. However, including background activity in the unified model improves goodness of fit for predicting individual spiking events. Uncovering the relationship between the model parameters and the timing of movements offers new ways to test hypotheses about the relationship between neural activity and behavior. We obtained significant spike-field onset time correlations from single trials using a previously published data set where significantly strong correlation was only obtained through trial averaging. We also found that unified models extracted a stronger relationship between neural response latency and trial-by-trial behavioral performance than existing models of neural information processing. Our results highlight the utility of the unified modeling framework for characterizing spike-LFP recordings obtained during behavioral performance.

## Introduction

Recordings of spike trains and local field potentials (LFPs) are increasingly becoming an important tool to study the spatiotemporal organization of information processing circuits underlying goal directed behavior (Pesaran et al., 2002, 2008; Nielsen et al., 2006; Buschman and Miller, 2007; Monosov et al., 2008). Studies have shown that neural information processing at each level of observation (spike, LFP, electroencephalogram) entails interaction of both evoked (input driven or stimulus related) and induced (background processes or intrinsic dynamics) factors (Arieli et al., 1996; Tallon-Baudry et al., 1998; Frien et al., 2000; Siegel and Konig, 2003). From an engineering perspective, any dynamic information processing system should have three important components: *ongoing background processes*, an *input* generated from external stimuli or cognitive event and an *output* which is generated by the interaction of input and the background processes. In this article we propose a family of parametric statistical models (unified models) for spike trains and LFPs that directly captures these features of neural information processing. The unified model family puts information extracted from spikes and fields on an equivalent statistical footing that can be statistically compared using existing decoding frameworks. Hence, the purpose of this article is two-fold. The first is to illustrate that using common parameters in unified models to capture spike-LFP data sets is useful for relating neuronal processing with goal-directed behavior. The second is to validate the performance of particular forms of spike-field models when using single trial decoding techniques (Banerjee et al., 2010).

Since spike occurrences are discrete events, conditional intensity processes are a natural choice to model neural signals (Commenges et al., 1986a,b). The spike count within a pre-defined window (which may be averaged across multiple trials) can represent how the unit processes information (Commenges et al., 1986a,b; Richmond and Optican, 1987; Britten et al., 1992, 1996; Hanes et al., 1995; Romo et al., 2002; Roitman and Shadlen, 2002; DiCarlo and Maunsell, 2005; Pouget et al., 2005; Monosov et al., 2008; Banerjee et al., 2010). Sensory stimuli can be encoded by different rates of neuronal spiking (Commenges et al., 1986a,b; Hanes et al., 1995; Pesaran et al., 2002; Monosov et al., 2008) in brain areas associated with stimulus processing. External events can also be decoded directly from the underlying spike rate (Richmond and Optican, 1987; Britten et al., 1992, 1996; Hanes et al., 1995; Romo et al., 2002; Wiener and Richmond, 2002). Understanding nervous system function from the perspective of neural encoding-decoding involves modeling of behavioral control variables and neural signals that dynamically evolve during goal-directed behavior. Often, including time-varying properties within a point process spike train modeling framework involves computing a time-varying rate function over an ensemble of trials (Richmond and Optican, 1987). Time-varying firing rate models have also been used to decode movement plans from single trials (Hanes et al., 1995; Wiener and Richmond, 2002). A similar statistical model for LFP activity has been developed in which a continuous Gaussian process is used to describe a time-varying signal with additive noise (Dawson, 1954; Emeric et al., 2008).

An underlying concern is that Poisson models do not contribute to high goodness of fit under standard significance thresholds (Truccolo et al., 2005; Banerjee et al., 2010). Analogously, a signal plus Gaussian noise model (SPN) cannot address the trial-by-trial variability in LFP signals (Truccolo et al., 2002). Existing studies in perceptual decision making (Horwitz and Newsome, 2001), somatosensory discrimination (de Lafuente and Romo, 2006), action selection (Cisek and Kalaska, 2005), and learning (Czanner et al., 2008) report single unit activity that varies trial-by-trial. In these cases variability in the neural signal does not disappear with additional training or further data collection, as the stimulus is designed to operate near the perceptual or cognitive decision making thresholds (Horwitz and Newsome, 2001; de Lafuente and Romo, 2006). One possible explanation is that trial-by-trial variability may not be generated by additive noise but rather by variability in preceding neural processing stages (Osborne et al., 2005). Thus, variability can have structured origins. To address structured sources of variability, trial-by-trial parameters have been introduced to scale the firing rate function (Bollimunta et al., 2007). Trial-by-trial parameters can also be introduced to scale the averaged evoked signal for LFP generation (Truccolo et al., 2002).

Introducing trial-by-trial parameters alone has important shortcomings, however. One problem is that simple trial-by-trial models do not take into account the autocorrelations in the neural time series due to interaction of incoming inputs and ongoing background activity. Physiological refractory periods can generate autocorrelations in the spike train time series as well as LFPs, leading to poor goodness of fit of spike-LFP models. Point process models provide a more realistic and accurate framework to decode information processing from spike trains at the single trial level (Daley and Vere-Jones, 1988; Barbieri et al., 2001; Kass and Ventura, 2001; Truccolo et al., 2005). These models have been used to relate the trial-by-trial non-stationarity in spike trains to ongoing background activity (Truccolo et al., 2005). The key component of this class of models is the inclusion of spike history as a parameter in the model. Multivariate extensions of these approaches have led to more sophisticated state space models to address neuronal firing at millisecond level precision (Srinivasan and Brown, 2007). State space models have two steps: defining a cognitive state as a lower dimensional representation of neural space (e.g., in multi-electrode recordings) and then studying the temporal evolution of these states (Yu et al., 2009). Defining the state variable depends on, amongst other factors, behavioral observations and statistical assumptions, which may be based on experimental design parameters set a priori rather than being purely data driven. Using generalized linear model (GLM) approaches, first order autocorrelations between events can be completely characterized and higher order autocorrelations can be approximated by choosing a higher model order.

Similar approaches have been used to model LFP dynamics and study the properties of large scale neural circuits (Brovelli et al., 2004). In the case of LFP activity, ongoing background activity can be modeled using linear autoregressive (AR) models in which continuous LFP activity can be expressed as a weighted sum of previous activity. A multivariate extension of such models (MVAR) on LFP activity has been used to study functional relationships between brain regions (Bollimunta et al., 2011). In this paper, we combine modeling of evoked responses and autocorrelated background activity in a unified framework that captures the role of stimulus-driven and background-driven information processing in spike train and LFP data. Model parameters can be fit through a combination of a systematic cross-validation scheme and Bayesian techniques. The modeling is computationally parsimonious and easy to implement. Moreover, these models can be readily applied to likelihood based decoding schemes to perform analysis at the level of single trials (Banerjee et al., 2010).

## Theory

### Unified generalized linear model (GLM) of spiking activity

Spike train observations are often modeled using the inhomogeneous Poisson process with a rate function λ(*t*) as the free parameter representing the mean rate of neuronal firing (Richmond and Optican, 1987; Hanes et al., 1995; Wiener and Richmond, 2002). This technique can be used to decode external events such as movement goals or decision making during cognitive tasks (Hanes et al., 1995; Wiener and Richmond, 2002). Rate models have been extended to variable rate models to explain neuronal firing trial-by-trial. Specifically, variable rate models remove the response latency jitters while calculating rate functions (Nawrot et al., 2003; Bollimunta et al., 2007). In the variable rate model, trial dependent rate function can be expressed as

(1) $$\lambda^{r}(t)=b^{r}\lambda_{0}(t-\tau^{r})$$

where λ^*r*^(*t*) is the rate at trial r with a trial invariant part λ_0_(*t*) scaled by amplitude *b*^*r*^ and lagged by latency τ^*r*^. We propose that studying these lags as functions of behavioral variables such as reaction times will reveal the information processing underlying complicated tasks. Suppose that the time-varying rate λ^*r*^(*t*) represents the instantaneous input to a unit and the observed spiking activity is the output during a trial. Equation (1) then becomes an information processing model for the neural activity. However, in this model the past spiking has no influence on the present spike. This is somewhat unrealistic as neurons are known to have refractory periods on the order of 10–20 ms or more (Truccolo et al., 2005). Hence, inclusion of spike history becomes important in modeling information processing from spike train observations.

Spiking activity can instead be modeled as a point process that relates the timing of a spike to past spike times (Daley and Vere-Jones, 1988; Barbieri et al., 2001; Kass and Ventura, 2001; Truccolo et al., 2005). In this framework, a discrete time-varying likelihood for spiking (conditional intensity) can be computed at millisecond resolution instead of a continuous rate function that expresses the firing rate. Mathematically, conditional intensity of spiking at a time *t* is expressed as

(2) $$\lambda (t|H_{t})=\underset{\Delta \to 0}{\lim}\frac{\mathrm{Pr}(N(t+\Delta )-N(t)=1|H_{t})}{\Delta }$$

where Pr(..|..) is the likelihood of spiking, conditioned on past history *H*_*t*_. The influence of background history can be expressed using the GLM framework (McCullagh and Nelder, 1989; Truccolo et al., 2005). Then, λ(*t*|*H*_*t*_) can be computed at the level of single trials and expressed as

(3) $$\lambda (t_{k}|H_{t_{k}})=\exp\text{​}\left(\gamma_{0}+\sum_{i\;=\;1}^{q}\gamma_{i}\Delta N_{t_{k}-i}\right)$$

where γ_0_ is the background firing rate and γ_*i*_'s are the constant coefficients that scale past spiking (Δ*N*_*t*_*k*__ = ∑^*k*^_*i* = 1_*N*_*t*_*i*__−∑^*k*−1^_*i* = 1_*N*_*t*_*i*__ = Δ*N*_*k*_, *N*_*t*_*i*__ is the spike count at *t*_*i*_).

To incorporate the information processing assumptions of input modulation and ongoing background activity in spike trains, we introduced the following conditional intensity function:

(4) $$\lambda (t_{k}|H_{t_{k}})=\lambda_{0}(t)\exp\text{​}\left(\sum_{i\;=\;1}^{q}\gamma_{i}\Delta N_{t_{k}-i}\right)$$

Thus, Equation (3) becomes a limit case scenario of Equation (4) in which the trial invariant firing rate is stationary. Finally, amplitude modulations and latency jitters can be incorporated within the conditional intensity by combining Equations (1) and (4) to address the effects of ongoing background activity and instantaneous input modulation to the neural dynamics on a trial-by-trial basis. According to this model, the conditional intensity λ(*t*|*H*_*t*_) is expressed as

(5) $$\lambda^{r}(t_{k}|H_{t_{k}})=b^{r}\lambda_{0}(t_{k}-\tau^{r})\exp\text{​}\left(\sum_{i\;=\;1}^{q}\gamma_{i}\Delta N_{t_{k}-i}\right)$$

where {γ} are the auto-regressive coefficients which weigh the spike history, Δ*N*_*t*_*k*__ is as defined for Equation (3), λ_0_(*t*) is the inhomogeneous Poisson firing rate that represents trial invariant instantaneous inputs, and *b*^*r*^ and τ^*r*^ are the amplitude scaling factors and lags that vary trial-by-trial. The trial varying parameters *b*^*r*^ and τ^*r*^ can be used to study ongoing behavior. The correlation of these parameters across multiple units with behavioral variables such as reaction times may reveal the functional connectivity underlying the task (Holdefer et al., 2000; Brugge et al., 2003; Lakatos et al., 2005; Shahaf et al., 2008).

### Estimation of spike model parameters

A Bayesian approach was used to estimate the parameters of the variable rate model (Bollimunta et al., 2007). Similar approaches have also been used to estimate model parameters in the Point process-GLM framework (Truccolo et al., 2005). We follow a combination of GLM-Bayesian parameter fitting to estimate the parameters of the unified model. First, the history coefficients (GLM parameters) are derived from a suitably chosen baseline epoch during which the firing rate can be considered stationary (Brody, 1999). Here, the spike train model is represented by Equation (2). Using the approach of Truccolo et al. (2005), the likelihood of spiking in one bin in a single trial is expressed as

(6) $$P(N_{t}{}_{{}_{1:K}}|\Theta )=\exp\left(\sum_{k=1}^{K}\log[\lambda (t_{k}|H_{t_{k}})\Delta ]\Delta N_{1:t_{k}}-\sum_{k\;=\;1}^{K}\lambda (t|H_{t_{k}})\Delta \right)+O(\Delta^{j})$$

where Θ = [{γ}, {*b*^*r*^}, λ(*t*|*H*_*t*_), {τ^*r*^}] is the parameter space of the unified model. The overall likelihood function can be computed over the ensemble of trials. The estimated GLM coefficients are those that maximize this overall likelihood function. We use the MATLAB (Mathworks Inc., Natick, MA) function “GLMFIT” to compute the GLM coefficients γ_*i*_ s, and the baseline firing rate exp(γ_0_). This code employs the iterative reweighted least squares (IRLSs) algorithm (McCullagh and Nelder, 1989).

Second, we estimate the trial varying parameters *b*^*r*^, τ^*r*^, and λ_0_(*t*) by following the approaches of Bollimunta et al. (2007). Taking the logarithm of the likelihood function in Equation (5) (*Q* = ln *P*), we obtain

(7) $$Q=\sum_{r=\;1}^{R}\sum_{k=1}^{K}\log[\lambda^{r}(t_{k}|H_{t_{k}})\Delta ]\Delta N_{1:t_{k}}-\sum_{r=\;1}^{R}\sum_{k=1}^{K}\lambda^{r}(t|H_{t_{k}})\Delta$$

Using Equation (2) we can further expand this expression,

(8) $$Q=\sum_{r=1}^{R}\sum_{k=1}^{K}\log[b^{r}\lambda_{0}(t_{k}-\tau^{r})\Delta ]\Delta N_{1:t_{k}}+\sum_{r=1}^{R}\sum_{k=1}^{K}\left(\sum_{i=1}^{q}\gamma_{i}\Delta N_{t_{k}-i}\right)\Delta \Delta N_{1:t_{k}}-\sum_{r=1}^{R}\sum_{k=1}^{K}\left[b^{r}\lambda_{0}(t_{k}-\tau^{r})\exp\left(\sum_{i=1}^{q}\gamma_{i}\Delta N_{t_{k}-i}\right)\right]\Delta$$

Taking the partial derivatives of *Q* with respect to λ_0_, *b*^*r*^, and setting them to zero, we get expressions for the maximum likelihood solution of each parameter. Finally, we obtain a iterative solution for τ^*r*^, λ_0_(*t*), and *b*^*r*^. The single trial latency shift at the *m* + 1 iteration is expressed as

(9) $$\tau_{m+1}^{r}=\arg\underset{\tau }{\max}\left(\sum_{t_{k=1}}^{K}\log[b^{r}\lambda_{0}(t_{k}-\tau^{r})\Delta ]\Delta N_{t_{k}}\right)-\sum_{k=1}^{K}\left[b^{r}\lambda_{0}(t_{k}-\tau^{r})\exp\left(\sum_{i=1}^{q}\gamma_{i}\Delta N_{t_{k}-i}\right)\right]\Delta$$

Thus, the algorithm begins with estimating the τ by maximizing the likelihood function.

(10) $$\lambda_{0}^{r}{\left(t\right)}_{m+1}=\frac{\sum_{r=1}^{R}\Delta N_{t_{k}+\tau_{m+1}^{r}}}{\sum_{r=1}^{R}b_{m}^{r}\exp\left(\sum_{i=1}^{q}\gamma_{i}\Delta N_{t_{k}+\tau_{m+1}^{r}-i}\right)}$$

Intuitively the numerator on the right hand side equals the spike count in a given time bin, computed after adjusting latencies of each individual spike train. Finally, the trial-by-trial amplitude scaling factors are computed by the following relation:

(11) $$b_{m+1}^{r}=\frac{\sum_{k=1}^{K}\Delta N_{t_{k}+\tau_{m+1}^{r}}}{\left[\sum_{k=1}^{K}\lambda_{0m+1}^{r}(t-\tau^{r})\exp\left(\sum_{i=1}^{q}\gamma_{i}\Delta N_{t_{k}+\tau_{m+1}^{r}-i}\right)\right]\Delta }$$

To ensure numerical stability, we constrained *b*^*r*^'s by (∑^*R*^_*r* = 1_*b*^*r*^)_*m* + 1_ = 1 at each iteration *m*.

It should be noted that for γ_*i*_ = 0, the unified model reduces to the Poisson model of spiking with trial-by-trial variability explored by Bollimunta et al. (2007). Also, in the absence of a strong time-varying external input, our model is equivalent to the simplest Point process spiking model explored by Truccolo et al. (2005).

### Unified autoregressive (AR) model for LFP activity

According to the signal plus noise model (SPN), all variability observed in single trials is accounted for by ongoing background noise (Truccolo et al., 2002). The event-related potential (ERP), time locked to a cognitive or perceptual event, is captured by the mean response. Thus, the signal *S*^*r*^(*t*) at time *t* and trial *r* is expressed as

(12) $$S^{r}(t)=I(t)+\xi^{r}(t)$$

where *I*(*t*) = 〈*S*^*r*^(*t*)〉_*r*_ is the time-varying mean evoked response, and ξ^*r*^(*t*) = *S*^*r*^(*t*)−〈*S*^*r*^(*t*)〉_*r*_ is the noise component with mean 0 and standard deviation σ. Hence, the parameter space for a model Θ can be expressed as {Θ : *I*(*t*), σ}. To capture the trial-by-trial variability of the evoked signal, a more general variable signal plus noise model (VSPN) was devised (Truccolo et al., 2002). In this model, LFP activity during a single trial is expressed as

(13) $$S^{r}(t)=b^{r}I(t-\tau^{r})+\xi^{r}(t)$$

where *b*^*r*^ and τ^*r*^ are the trial-by-trial amplitude and latency jitters.

From an information processing perspective, both SPN and VSPN models capture the interaction of external input and ongoing background activity with a simple linear sum. However, the white noise component in the model is not biologically realistic and does not capture the presence of induced activity (Tallon-Baudry et al., 1998; Frien et al., 2000). To capture induced activity as a contributor to information processing, we introduced the unified AR model for ongoing background activity. Here, we do not assume the independence of observations at successive time points. Instead, we consider that an instantaneous input from earlier processing stage *I*(*t*) dynamically interacts with ongoing background neural activity in the brain region in which LFP recordings are obtained. In other words, the LFP activity at a certain time point is a function of the instantaneous input and the ongoing background activity, which is influenced in turn by past activity. Mathematically, the measured neural activity *S*(*t*) at time *t* will depend on the instantaneous input *I*(*t*) and neural activity from the past, *S*(*t*−*i*) up to an order *I* = *p* (Percival and Walden, 1993; Ding et al., 2000). The neural signal at time *t* is then expressed as

(14) $$\begin{array}{cc} S^{r}(t)=\sum_{i=1}^{p}a_{i}S^{r}(t-i)+b_{r}I(t-\tau_{r})+\xi^{r}(t) & \xi^{r}\forall N(0,\sigma^{2}) \end{array}$$

where *b*_*r*_ and τ_*r*_ are terms to account for the trial-by-trial amplitude and latency variability of the input, *r* is the trial index, and ξ^*r*^ is the residual white noise with zero mean and standard deviation σ. Hence, the parameter space of the unified model is expressed as {Θ : *a*_*i*_, *p*, *b*_*r*_, τ_*r*_, *I*(*t*), σ}. Like the GLM formulation of spike trains, the unified model of LFP background activity captures both the evoked and induced components of neural activity.

### Estimation of model parameters

The parameters of the VSPN model are estimated using a Bayesian approach (Truccolo et al., 2002). Along similar lines, we estimate the model parameters of the unified model in two steps. In the first step, the ongoing background parameters [*a*_*i*_ in Equation (14)] are computed from a baseline condition. The AR coefficients (*a*_*i*_) can be obtained by applying Burg's algorithm (Percival and Walden, 1993; Ding et al., 2000) to the mean removed training set data (pre-stimulus baseline activity) *X*(*t*) = *S*(*t*)− < *S*(*t*) >. The model order p can be obtained by using the Akaike information criterion (AIC; Akaike, 1973). For the observed data, the stimulus related evoked input *I*(*t*) can then be expressed as

(15) $$I^{\text{test},r}(t)=S^{r}(t)-\sum_{i=1}^{p}a_{i}S^{r}(t-i)$$

In the second step, we use Bayesian techniques to compute the trial-by-trial amplitude (*b*_*r*_) and latency variability (τ_*r*_) in Equation (14), which effectively reduces to fitting *b*_*r*_, τ_*r*_ and *I*(*t*−τ_*r*_) to the following equation

(16) $$I^{\text{test},r}(t)=b_{r}I(t-\tau_{r})$$

The posterior (joint probability distribution) for the model parameters is expressed as

(17) $$P(I,\{b^{r}\},\{\tau^{r}\}|I^{\text{test},r},G)=\frac{P(I^{\text{test},r}|I,\{b^{r}\},\{\tau^{r}\},G)P(I,\{b^{r}\},\{\tau^{r}\}|G)}{P(I^{\text{test},r}|G)}$$

Thus, the likelihood of the data for single trial given the model parameters is expressed as

(18) $$P(I^{\text{test},r}|I,\{b^{r}\},\{\tau^{r}\},G)=\frac{1}{\sqrt{2\pi \sigma^{2}}}\exp\left\{-\frac{{\left(I^{\text{test},r}-b^{r}I_{t-\tau^{r}}\right)}^{2}}{2\sigma^{2}}\right\}$$

where, G is the prior information, and the residual (ξ) follows a Gaussian distribution across trials. We also assume the shape of the distributions for *b*^*r*^ and τ^*r*^ as uniform with a known range of values: [0, 1] for *b*^*r*^ and [−30, 30] ms for τ^*r*^ with the following constraints

(19) $$\begin{array}{l} P(I|G)=Const\\ P(\{\tau^{r}\}|G)=Const\\ P(\{b^{r}\}|G)=Const \end{array}$$

The likelihood of the entire data can be expressed as the product of the likelihood for all trials and observations.

(20) $$P(I^{\text{test},r}|I,\{b^{r}\},\{\tau^{r}\},G)=\prod_{r=1}^{R}\prod_{t=1}^{T}P(I_{t}^{\text{test},r}|I_{t},b^{r},\tau^{r},G)$$

Substituting Equation (18) in (20) and using (17) and (19) we obtain the posterior for the model given data in all trials.

(21) $$P(I^{\text{test},r}|I,\{b^{r}\},\{\tau^{r}\},G)\propto \exp\left\{-\sum_{r=1}^{R}\sum_{t=1}^{T}\frac{{\left(b^{r}I_{t-\tau^{r}}-I_{t}^{\text{test},r}\right)}^{2}}{2\sigma^{2}}\right\}$$

By taking the logarithms of both sides, we obtain

(22) $$\ln P\propto -\sum_{r=1}^{R}\sum_{t=1}^{T}\frac{{\left(b^{r}I_{t-\tau^{r}}-I_{t}^{\text{test},r}\right)}^{2}}{2\sigma^{2}}$$

The model parameters *b*^*r*^, τ^*r*^, and *I*_*t*_ can be obtained by maximizing Equation (22) iteratively. Thus, we obtain the expressions for *I*_*t*_, *b*^*r*^ and τ^*r*^.

(23) $$\tau^{r}=\arg\underset{\tau }{\min}\sum_{t=1}^{T}{\left(b^{r}I_{t-\tau^{r}}-I_{t}^{\text{test},r}\right)}^{2}$$

(24) $$I_{t}=\frac{\sum_{r=1}^{R}I_{t+\tau^{r}}^{\text{test},r}}{\sum_{r=1}^{R}b^{r}}$$

(25) $$b^{r}=\frac{\sum_{t=1}^{T}I_{t+\tau^{r}}^{\text{test},r}}{\sum_{t=1}^{T}I_{t}}$$

## Experimental methods

Neural data in this study were collected from two adult male rhesus macaques. All surgical and animal care procedures were done in accordance with the National Institute of Health guidelines and are approved by the New York University Animal Care and Use Committee.

### Training and surgical procedures

Monkeys were first trained to sit in a custom chair and make visually guided reaches to a touch-sensitive screen (ELO Touch Systems, CA) mounted in front of either a LCD display or a custom LED board on which targets were presented. Behavior was controlled using custom Labview (National Instruments, TX) code running on a real-time PXI platform. After several weeks of training, a head restraining prosthesis was implanted on the skull using metal and/or ceramic screws and dental acrylic to maintain stable head position during recordings. All procedures were done under isoflurane anesthesia and sterile conditions. At least 3 weeks after surgery, each monkey began training in eye movement tasks as well as reaching tasks. Eye position was monitored with an optical video eye tracker (ISCAN, Cambridge, MA).

Once an animal was proficient at making saccades and saccades with reaches on command, a second surgery was performed in order to implant a recording chamber over the posterior parietal cortex of one hemisphere. Stereotaxic surgical coordinates and structural magnetic resonance imaging were used to identify the position of the intraparietal sulcus and to guide placement of the recording chamber. After a week of recovery, 1 MΩ tungsten electrodes (Alpha Omega, Israel) could then be used to record single unit, multiunit and LFP activity while each subject performed reaches and saccades. Up to eight electrodes at a time were lowered into the brain using the Double MT system from Alpha Omega—4 in the lateral intraparietal area (LIP) and 4 in the parietal reach region (PRR).

### Behavioral tasks

The LED stimulus board or LCD display was placed behind a glass touch screen oriented in a vertical plane in front of the monkey so that eye and hand movements were made in the same visual workspace. Each trial was initiated when a monkey touched a pair of proximity sensors placed near the body with his hands. The non-reaching hand was required to maintain touch on a proximity sensor throughout the trial. Access to water was controlled during training and testing, and each animal was habituated to head restraint and trained to perform oculomotor and reaching tasks for a liquid reward. A brief auditory tone preceding reward delivery served as a secondary reinforcer on all correct trials. For neural recordings, reaches were made with the hand contralateral to the hemisphere under study.

In experiment 1, a trial started with the illumination of a central red and green stimulus. Monkey A performed a cued look-and-reach reaction time (RRT) task. He fixated and touched the central targets and waited 300–400 ms for cue instructions. Following this period, a red, green, or yellow effector cue appeared just above the central fixation, cuing the monkey to plan a saccade, reach, or combined saccade and reach, respectively. After a 500–700 ms baseline period, a blue or white instruction LED was illuminated next to the effector cue. After an additional 500–700 ms, the touch, fixation, effector cue, and instruction cues were extinguished and two peripheral cues, one blue and one white, appeared briefly (200–300 ms) 10° to the left and right of fixation. The monkey was then required to make the cued movement to the remembered location of the cued target. Trials were aborted if the saccade did not reach the target within 700 ms and reaches within 900 ms. Finally, 150–250 ms after target acquisition, the target reappeared for a 300–400 ms hold period, after which the monkey was given a fluid reward.

In experiment 2, monkey B performed a memory-guided saccade task involving a saccade from a central location to a peripheral target. The trial started with the illumination of a central red and green stimulus. The monkey fixated and touched the central target location and waited 500–800 ms. Subsequently, a peripheral red target was flashed for 100 ms, followed by a delay of 1000–1500 ms. The monkey was then cued through extinction of the central red fixation target to saccade to the remembered position of the red target. The monkey waited until the go signal for each movement before promptly making that movement and had to respond to the go signal with a saccade within 350 ms. To prevent the monkey from trying to predict the go signal, trials were aborted if the saccade reaction time (SRT) was less than 100 ms. After holding the target successfully for 300–500 ms, the monkey was given a liquid reward.

### Data collection and preprocessing

Eye position and touch position on the screen were sampled at 1 kHz. Each signal was time-stamped and streamed to disk along with data about each trial from the LabVIEW behavioral control program. The time of cue presentation was recorded as the time at which a photosensor detected a simultaneous stimulus change on the monitor. Spiking and LFP activity were recorded with 1 MΩ tungsten electrodes (Alpha Omega, Nazareth Illit, Israel). Neural signals were amplified (×10,000; TDT Electronics, Alachua, FL), digitized at 20 kHz (National Instruments), and continuously streamed to disk during the experiment (custom C and Matlab code). LFP activity was generated by low-pass filtering the raw, broad-band recording at 300 Hz and decimating the signal to 1 kHz. Single-unit activity was generated by band-pass filtering the signal from 0.3 to 6.6 kHz, extracting waveforms that crossed a threshold (typically 3.5 SDs of the band-pass filtered activity), up-sampling and aligning waveforms to the peak negativity or positivity, and semi-automatically clustering waveforms on a 100 s moving window. Typically, spike clusters were over-clustered automatically and then manually inspected and merged.

## Results

We first present the results of applying our proposed unified modeling framework to simulated spike trains and LFP and analyze the accuracy and sensitivity of fitting trial-by-trial parameters in comparison with variable signal models (variable rate for spikes and VSPN for LFPs; Truccolo et al., 2002; Bollimunta et al., 2007). We then present the results of applying unified models, the variable signal models and, for further comparison, noise models (rate model for spikes and SPN model for LFPs) on spike-field recordings from two different experiments involving alert macaque monkeys. We also present the results of application of the accumulated likelihood framework (AccLLR; Banerjee et al., 2010) to decode target selection times (STs) from experiment 1 and visual response onset times in experiment 2. In the remainder of this article, we refer to the SPN model for LFPs and the rate model for spikes together as noise models. Similarly, we refer to the VSPN and variable rate models as variable signal models when discussing spike-field models. While discussing these models in the context of each individual recording (spikes or LFPs), we use the names prevalent in the literature (Truccolo et al., 2002; Bollimunta et al., 2007).

### Simulated spike trains

To produce simulated spike trains, the trial invariant rate function λ(*t*) of Equation (5) was chosen to be a Gaussian function (Bollimunta et al., 2007) centered at 750 ms with a trial duration of 1500 ms and a constant background firing rate B.

(26) $$\lambda_{0}(t)=B+\frac{1}{\sqrt{2\pi }\sigma }\exp\left(-\frac{{\left(t-750\right)}^{2}}{2\sigma^{2}}\right)$$

where *B* = 2 Hz and σ = 80 ms. The latency lags τ^*r*^ were sampled from a uniform distribution in the interval [−200, 200] ms. The amplitude scaling factors were sampled from a uniform distribution in the interval [15, 25]. Realistic history coefficients were chosen to generate spiking activity using a point process-GLM model by modeling spike train observations in the LIP of a macaque monkey performing simple reaction time tasks (see Application to experimental data). We generated spiking activity using Ogata's thinning method (Ogata, 1981) with the conditional intensity function expressed in Equation (5).

**Figure 1:**
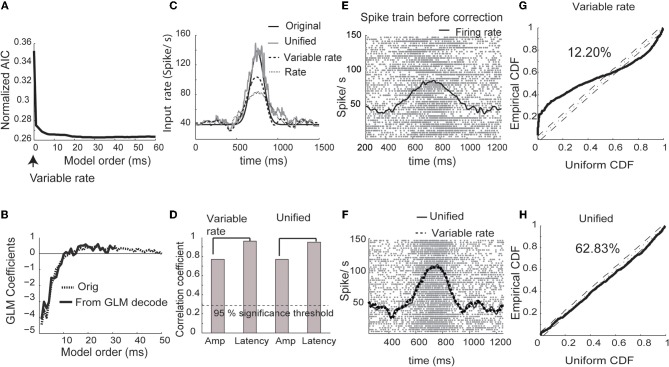
**Simulated spike trains. (A)** Normalized Akaike information criterion (AIC) versus unified model order. The optimum model order corresponds to the minima of the AIC curve. **(B)** The original GLM coefficients used for generation of simulated data and the estimated coefficients of the unified model (minimum of AIC curve in **A**). **(C)** Estimated input using the rate model, the variable signal model (Bollimunta et al., 2007) and the unified model. **(D)** Correlation coefficients obtained between original and estimated amplitudes and latency from the variable signal and unified models respectively. **(E)** Spike rasters for 50 test trials and the mean firing rate (black trace). **(F)** Spike rasters corrected using the unified model. Mean corrected firing rates are plotted using unified (solid trace) and variable signal (dashed trace) models. **(G)** CDF of simulated inter-spike intervals rescaled using the conditional intensity function for the variability only model plotted against the CDF of uniform distribution (solid line). The dotted line in the center shows the cumulative density function of uniform distributions. The dashed lines in parallel reflect the boundaries at 95% significance level. The percentage reflects the ratio of spikes captured by the model within 95% significance. **(H)** Same plot as G plotted for unified model.

We estimated the history coefficients from a baseline period [1, 500] ms during randomly chosen training trials (*n* = 50) in which the input was considered to be non-fluctuating in amplitude and latency. The model order was selected according to the AIC (Akaike, 1973). Bayesian iterative algorithm (Equations 9–11) was then used to estimate the input, trial-by-trial amplitude and latency fluctuations. We present the results of the simulation and estimation of information processing parameters in Figure 1. The initial values of amplitude modulation in each trial were set to one and latency fluctuations to zero. Improvement in the quality of estimated instantaneous rate function was evaluated at the end of the each iteration using a least squares measure. If estimate quality improved from one iteration to the next by more than 1%, the algorithm was continued. Otherwise, the rate function at the latest iteration was accepted as the solution. For all practical purposes, the iteration number never went beyond five (see Methods for details). The AIC was lower for the unified model parameters than for the variability model (Figure 1A). The estimated GLM parameters weighting the spike history matched the one used for simulation (Figure 1B).

For comparison, we estimated trial-by-trial and trial-invariant rate parameters using the variable rate model (Bollimunta et al., 2007). The input rate estimated using the unified model was close to the real input rate that was used for simulating the spike trains (Figure 1C). The unified model came closest to estimating the original input rate, followed by the variable rate model and the rate model. Both the unified model and the variable rate model (Bollimunta et al., 2007) yielded similar results for the amplitude and latency estimation (Figure 1D). Spike trains before and after amplitude and latency correction using each modeling strategy are plotted in Figures 1E and F, respectively. Again, the unified model and the variable rate model showed drastic improvements in the estimation of firing rates. In order to quantify the goodness of fit from unified and variable rate models, we plot the cumulative distribution function (CDF) of the inter-spike intervals (ISIs) rescaled by the time-rescaling theorem (Brown et al., 2002) against the CDF of a uniform distribution between 0 and 1 (Figures 1G,H). If all spike occurrences are captured in these plots, each data point should be on the 45° diagonal line. Thus, goodness of fit for reconstructing observed spikes using a model can be quantified around a confidence interval bounding this line. Within 95% confidence intervals, 63% of spikes are captured by the unified model compared to 12% by the variable rate model.

**Figure 2:**
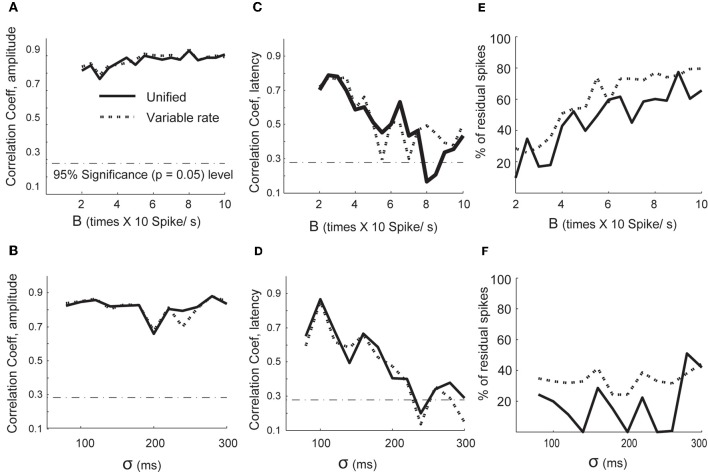
**Performance of spike model parameter estimation when varying signal and noise scenarios.** Correlation coefficients between the estimated and original parameters are plotted on the y axis in **(A–D)**. **(A)** Changes in correlation coefficients between estimated and original amplitude scaling factors with change in **(B)** (background firing, Equation 26) and **(B)** Changes in correlation coefficients between estimated and original amplitude scaling factors with change in σ (signal width). **(C)** Changes in correlation coefficients between estimated and original lags with **(B,D)** σ. **(E)** The percentage of spikes unaccounted by the conditional intensity function of variable signal and unified model as a function of **(B)**. **(F)** The percentage of spikes unaccounted by the conditional intensity function of the variable signal and unified model as a function of σ.

We analyzed the performance of both the variable rate and unified models under varying signal to noise scenarios (see Figures 2A–F). The performance for amplitude estimation was relatively independent of SNR changes introduced by a baseline (B) increase in the firing rate and strength of the signal (σ values; Figures 2A,B). The estimation of latency lags was adversely affected for both B and σ values (Figures 2C,D). We also computed how varying signal and noise strengths affect the goodness of fit of the spike train models, as shown in Figures 2E,F. We computed the percentage of points captured by the slope of empirical CDF plotted against the CDF of a theoretical uniform distribution within 95% confidence level at each signal and noise strengths. Overall, we found fewer spike occurrences were unaccounted for by the unified model compared to the variable rate models for most values of baseline firing rate (B) and width of the mean rate (σ; Figures 2E,F). In the higher noise to signal scenario, the performance of both families of models deteriorated. Thus, the parameters for the unified model can be estimated with quantifiable degree of sensitivity, and the signal-to-noise ratio needs to be considered when applying either of the modeling techniques.

### Simulated local field potentials

We simulated representative LFP time series using a linear AR Gaussian model. The driving input waveform was chosen to be a mixture of Gaussian functions that peaked at 700 and 800 ms.

(27) $$I(t)=\exp\left(-\frac{{\left(t-700\right)}^{2}}{2\gamma^{2}}\right)-\exp\left(-\frac{{\left(t-800\right)}^{2}}{2\gamma^{2}}\right)$$

The inputs were scaled by trial-by-trial amplitude scaling factors and shifted in time with trial-by-trial latency lags. The values of the amplitude scaling factors and latency lags at each trial were sampled from uniform distributions: [0, 2] and [−100, 100], respectively. The AR coefficients used for this simulation were derived from fitting experimental recordings of neural data with an AR model (see Application on neural data) to make them realistic. Using the input waveform, AR coefficients and white noise with standard deviation 0.15 (to mimic the physiological noise to signal ratio), we generated 200 trials of LFP with 1500 ms duration.

**Figure 3:**
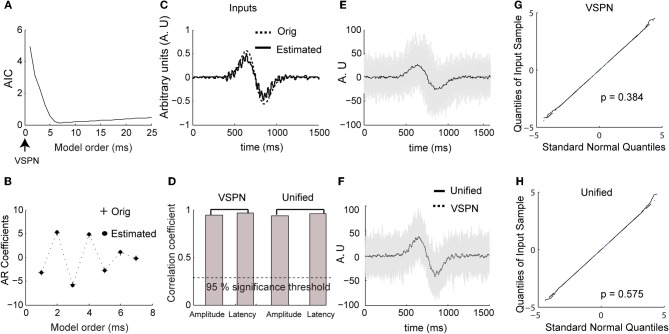
**Simulated LFP data. (A)** Normalized Akaike information criterion (AIC) against unified model order. The optimum model order corresponds to the minima of the AIC curve. **(B)** The original AR coefficients (+) used to generate simulated data and the estimated coefficients (solid dots) of the unified model (corresponding to minimum of AIC curve in **(A)**. **(C)** The original (dashed) and estimated (solid) input to unified model. **(D)** Correlation coefficient between original amplitude and estimated amplitude, original latency and estimated latency for VSPN and unified model. The dashed line below shows the value of correlation coefficient at *p* = 0.05 level. **(E)** Traces of simulated LFP data (gray) and averaged LFP (black) across all trials. **(F)** Traces of corrected LFP data following the unified model. **(G)** Quantiles of residual time series obtained from modeling with VSPN model plotted against standard normal quantiles. The *p*-value is from the Kolmogorov–Smirnov test on residual data against the null hypothesis that they come from a normal distribution. **(H)** Same as **(G)** but performed on residuals of unified model.

We estimated AR coefficients using Burg's algorithm (Percival and Walden, 1993) on ensemble mean removed LFP time series data from the 100 ms segment of time at the start of each trial in which no stimuli were presented or behaviors required. The optimum model order (number of AR coefficients) was computed using AIC (Figure 3A; Akaike, 1973). The model order (complexity) contributing to the minimum AIC was considered to be the optimal (Ding et al., 2000). Figure 3B presents the values of the original AR coefficients used in the model and shows that they are almost identical to the estimated AR coefficients. We applied the VSPN model of Truccolo et al. (2002) and the unified model to estimate the input, amplitude and latency. Both models yielded equivalent results for estimation of amplitude and latency factors. However, the shape of the estimated input could be accurately computed only by the unified model (Figures 3C,D). As presented earlier for spiking activity, we plot the original (Figure 3E) and amplitude-latency corrected (each trial was shifted by the estimated latency τ and scaled by the amplitude scaling factor *b*) LFP signals (Figure 3F) from the unified model. Again, the mean responses from the unified model and the VSPN model matched very closely (Figure 3F). We computed the goodness of fit of each model using the Kolmogorov–Smirnov (K–S) test on the residual data against the null hypothesis that the residuals come from a standard normal distribution. The *p*-value expresses the proximity of the residual data to the standard normal distribution. We plot the quantiles of the residual data against the quantiles of the standard normal distribution (Figures 3G,H). We obtained *p* = 0.58 for the unified model and *p* = 0.38 for the VSPN model. In both cases the null hypothesis could not be rejected at *p* = 0.05 level and hence, residuals can be considered as normally distributed.

**Figure 4:**
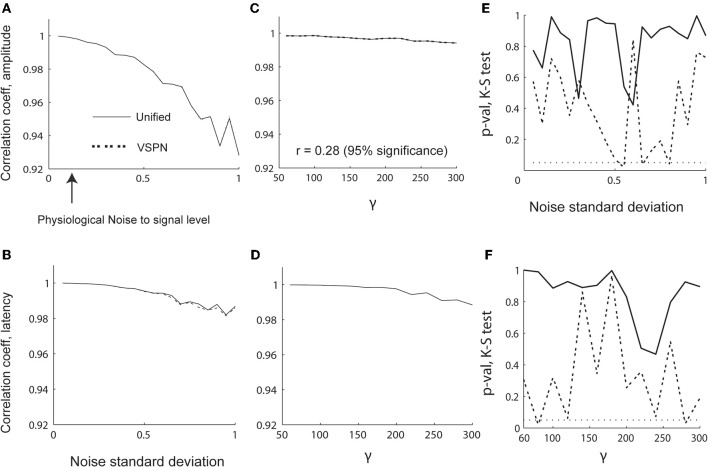
**Performance of LFP model parameter estimation in varying signal and noise scenarios. (A–D)** Correlation coefficients between estimated and original parameters. Changes in correlation coefficients between estimated and original **(A)** amplitude scaling factors and **(B)** latency lags with varying noise standard deviation (σ). Changes in correlation coefficients between estimated and original **(C)** amplitude scaling factors and **(D)** latency lags with varying γ (signal width). The *p*-values computed using K–S test performed on residual LFP traces from VSPN and unified modeling framework as functions of σ **(E)** and γ **(F)** respectively.

To evaluate the sensitivity of model parameter estimation, we varied the strength of the signal [γ in Equation (27)] from 60 to 300 in steps of 20 while keeping the standard deviation of the noise [σ in Equation (14)] fixed at 0.15. We also varied σ from 0.05 to 1 in steps of 0.05 while keeping γ = 100. Thus, we could test the quality of the model parameter estimation under different signal to noise ratios. We plot the correlations between the estimated and original model parameters in Figures 4A–D. Unlike parameter estimations using the unified and variable rate models for spikes, the unified and VSPN model parameter estimation for LFPs did not deteriorate as much with amplitude scaling factors and lags. The results of VSPN and unified models are similar with a very small dip in correlation coefficients in higher noise to signal ratio scenarios. We tested the residuals for each σ and γ scenario with K–S tests of goodness-of fit and plotted the *p*-values of the null hypothesis in Figures 4E,F. We observe larger *p*-values for the unified model residuals compared to VSPN model residuals, particularly at higher noise to signal scenarios (higher values of γ and σ). In total, the residuals of the unified model yielded *p* > 0.05 for all σ and γ configurations. For the VSPN model, this occurs 85% of the time, indicating that the unified model performs better than the VSPN model in capturing the LFP data. However, the two models yield similar results for amplitude and latency estimation.

### Cued look-and-reach reaction time task

**Figure 5:**
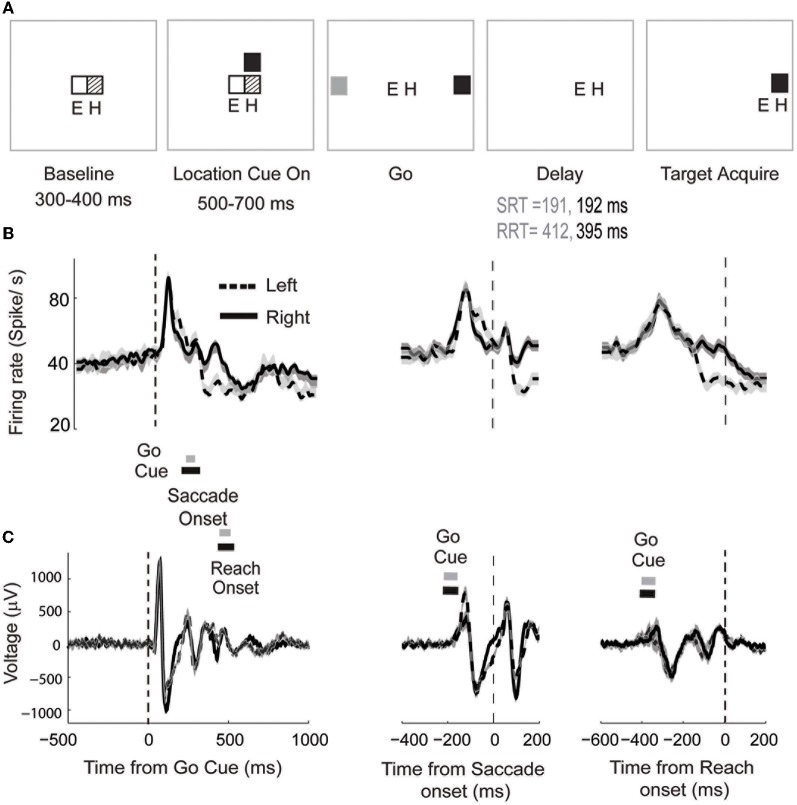
**The cued look-and-reach reaction time task. (A)** Sequential steps (trial starts from the left) with which the stimuli are presented. Eye and hand positions are marked with E and H respectively (for details, see text). **(B)** Mean firing rates for leftward (dashed) and rightward (solid) movement smoothed with a 5ms Gaussian window. The shaded area indicates 95% confidence interval. Mean ± standard deviation of saccade and reach onset times are shown for leftward movements (gray bars) and rightward movements (black bars). Data is aligned to Go Cue (left panel), saccade onset (middle panel) or reach onset (right panel). The mean time of the Go Cue is shown for leftward movements (gray bars) and rightward movements (black bars). **(C)** Averaged LFP responses simultaneously recorded with the spiking activity.

We demonstrate the use of parametric models for trial-by-trial analysis of neural signals in an example data set in which spike-LFP recordings in LIP were made during the performance of a behavioral task. Monkey A performed a cued look-and-reach task. The details of the task are described in the Experimental Methods and shown in Figure 5A. In this experiment, we studied the neural activity recorded from 500 ms before the onset of the Go cue to 1000 ms following cue onset. The Go cue was the signal cuing the monkey to make a simultaneous saccade and reach to a spatial target with the hand contralateral to the hemisphere of our recordings (see Experimental Methods for details). We estimated averaged neural responses by time locking the spike trains and LFP to different behavioral events, e.g., Go cue, saccade onset and reach onset, and computing the corresponding firing rate functions (Richmond and Optican, 1987; see Figures 5B,C for details). Our goal was to determine if the target STs decoded by using the unified model and the two other models in the literature were correlated trial-by-trial with the reaction time. The three behavioral events and underlying neural responses of prime interest were: (1) the time at which the cue to make a movement appeared (Go cue), (2) the time at which the saccadic eye movement began (saccade onset) and (3) the time at which the arm began reaching towards the target (reach onset). The time from Go cue to saccade onset and reach onset were defined as the SRT (see Figure 5) and RRT, respectively. In a related study, we found that correlation between SRT and RRT can be a measure of eye-hand coordination (Dean et al., 2011). In the current data set, we find significant correlation between SRT and RRT for both reaches to the left (*r* = 0.29, *p* < 0.01) and reaches to the right (*r* = 0.28, *p* < 0.01).

**Figure 6:**
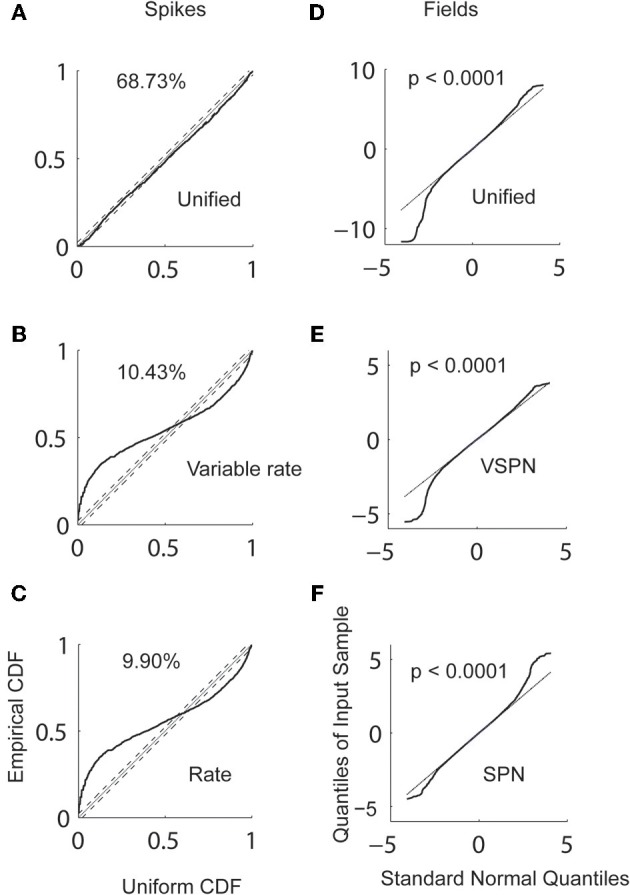
**Goodness of fit analysis.** The empirical cumulative density function (CDF) of rescaled inter-spike intervals using the conditional intensity of **(A)** unified **(B)** variable rate and **(C)** rate models are plotted against the CDF of a uniform distribution. The percentages of points in empirical CDF that falls within 95% confidence bound (dashed lines) of the theoretical CDF (dotted line) are reported. The quantiles of residuals LFPs from **(D)** unified **(E)** VSPN and **(F)** SPN models plotted against the quantiles of standard normal distribution. The *p*-values of K–S tests performed on the residual with the null hypothesis that the residual distribution is normal are reported.

Estimating the unified model parameters for spike trains began with computation of the GLM coefficients (Equation 6). We computed the GLM coefficients within a window of [−500, 0] ms aligned on the Go cue using data from 100 randomly chosen trials out of a sample of 176 trials (82 trials into the response field and 94 trials out of the response field). Thus, comparable samples of trials from both directions of movement were used to compute the GLM coefficients. The optimum GLM order 50 was chosen using the AIC (Akaike, 1973; Truccolo et al., 2005). Next, we computed the trial-by-trial amplitude scaling factors and the latency jitters for the conditional intensity function using the Bayesian iterative procedure (Equations 9–11) with a time window that spanned from the Go cue to saccade onset. For comparison, we also fitted the spike trains with the variable rate model (Bollimunta et al., 2007) as well as the inhomogeneous Poisson firing rate model (also referred to as the rate model) with no trial-by-trial variability or history within the same time window [Go cue, SRT] for both directions of movement. We plot the empirical CDF of the ISIs after rescaling time according to the fitted condition intensity function from a spike train model against the CDF of a uniform distribution (Figure 6). For movements into the response field, the unified model captures 69% of spikes within a 95% confidence level, compared to 10% of spikes by the variable rate model and 10% by the rate model (Figure 6). Similar results are observed for movements out of the response field (not shown here).

We also reconstructed LFPs using the estimated unified model, the VSPN model and the SPN model parameters. We fit the AR coefficients of the unified model using Burg's algorithm applied to the LFP data within a time window of [−500, 0] ms aligned to the Go cue. The trial-by-trial amplitude scaling factors and lags were estimated using a Bayesian iterative algorithm (Equations 23–25) within the event interval between the Go cue and the maximum SRT (257 ms). As with spikes, LFPs were reconstructed using the VSPN model and the SPN model of LFP activity. The goodness of fit of each reconstruction was computed by performing non-parametric K–S tests on residual data. In Figure 6, we plot the quantiles of residual data against the quantiles of standard normal data. A K–S test on the residuals shows that the distribution of the residuals is significantly different from a normal distribution (*p* < 0.0001) for both movement directions in all three models. Therefore, none of the models results in normally distributed residuals for this LFP data set. In the future, one way to improve the unified model might be to make the AR coefficients dynamic. However, any increase in the dimensionality of the parameter space needs to be cross-validated. Another way to address this is by incorporating task relevant covariates (Truccolo et al., 2005; Czanner et al., 2008). Both of these approaches require additional parameter fitting and lie outside the scope of discussion of the present article.

**Figure 7:**
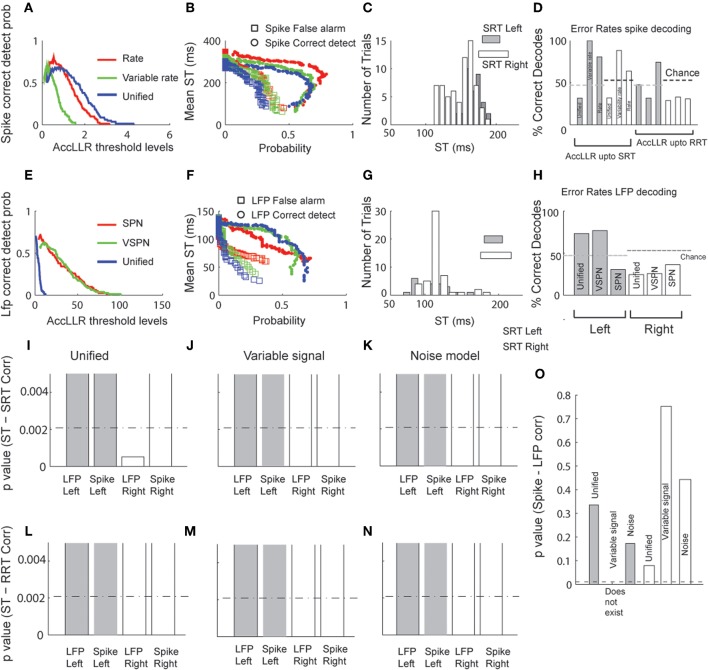
**Decoding of target selection times (ST) via AccLLR analysis. (A)** Correct detect probabilities for leftward movements as a function of AccLLR levels computed using the unified model (blue), the VSPN model (green) and the SPN (red) model of LFP activity. The inverse of correct detect probability is the error rate. **(B)** Mean ST (from all correct decodes) obtained from each model for detecting movements to the left at varying false alarm (squares) and correct detect probabilities (circles) (see text for details). **(C)** Histograms of trial-by-trial ST for leftward and rightward movements. For direct comparison with behavior, mean SRT and RRT are shown for left (gray) and right (white) movement directions. **(D)** The error rates in decoding the correct direction (leftward or rightward) of movement from LFP activity. **(E)** Same as in **(A)**, now computed for unified model (blue), variable rate model (green) and rate model (red) of spike trains. **(F)** Mean target selection time (ST) as a function of probabilities as in **(B)**, computed using the three spike train models. **(G)** Trial-by-trial selection times decoded from spike trains for left and right movement directions. Conventions as in **(C)**. **(H)** Error rates for spike trains. Since accumulation of log-likelihood ratios up to SRT could not perform better than chance for variable rate and the rate models, we allowed accumulation up to RRT. For comparison of the results from different models/movement directions we present **(I–K)** *p*-values of correlation between SRT and selection times from unified **(I)**, variable rate **(J)** and rate **(K)** models respectively. Bonferroni correction thresholds at *p* = 0.05 (*N* = 24) are plotted in dashed lines. The bars are clipped at *p* = 0.005 to highlight smaller values (significant region of interest). **(L–N)** Same as in **(I–K)**, except SRT replaced by RRT. **(O)** *p*-values of Spike-LFP selection time correlations. None of the correlations were significant. Bonferroni correction thresholds for *p* = 0.05 (*N* = 5) are plotted in dashed lines.

We applied the AccLLR decoding framework (Banerjee et al., 2010) to estimate target STs from the LFPs and the spike trains trial-by-trial. We defined two alternative models for ST discrimination: a model for movements to the left and a model for movements to the right. Each alternative model in the AccLLR framework can be defined by the unified model, a variability signal (variable rate and VSPN) model or a noise model (rate model for spike trains and Gaussian/SPN model for LFP) of neural activity. If the accumulated log-likelihood ratio for a leftward trial hits the sequential probability ratio test (SPRT/AccLLR) threshold for leftward movement decoding, correct detection occurs (true positive; Wald and Wolfowitz, 1947). On the other hand, if accumulation hits the rightward AccLLR threshold (for the alternative model), an error in decoding of correct direction occurs (false positive). We have previously shown how controlling the false alarm rate is an important part of any decoding of neural signals, particularly in measuring spike-field correlations, because the speed-accuracy trade off in STs is governed by these rates (Banerjee et al., 2010). In Figure 7, we present the detailed results of the decoding performance using the three different modeling procedures for comparison.

In Figure 7A, we plot correct detect probabilities (probability of hitting a correct AccLLR threshold) for decoding movement direction from spikes versus varying levels of AccLLR threshold (see Banerjee et al., 2010 for details) for each model. The maximum correct detection (hit) probability is comparable for each model. We computed the mean ST for movements out of the response field and plot the ST versus the probability of correct detection (hit) or false alarm (Figure 7B). Since the correct detect probability falls off around a peak value (Figure 7A), two different mean STs can correspond to a single value of correct detect probability. The unified model always had lower false alarm rates than the variable rate model and the rate model. In Figure 7C, we show a histogram of the single trial LFP STs using the unified model for trials before each movement direction. Here, we set the AccLLR threshold such that the false alarm probability was less than the probability of detecting true negatives. We used the trials with movements out of the response field to set the threshold for the trials with movements into the response field and vice versa. The significance of error rates were tested with chi-squared statistics with the null hypothesis that the error rate over a sample was obtained by chance (random assignment of direction to a trial). Using the unified model resulted in lower error rates than using the variable rate model or the rate model (Figure 7D) if we set the maximum time for ST detection in each trial to the SRT. The decoding performance was significantly lower than chance for movements out of the response field for the unified model (χ^2^(1) = 5.66, *p* < 0.05) but not for the variable rate model or the rate model (χ^2^(1) < 3.84, *p* > 0.05 for each direction and model type, Figure 7D). If the maximum time to ST detection in each trial was allowed to extend up to the RRT, which was typically longer than SRT (mean difference between SRT-RRT: 221ms for left and 203ms for right), the error rates from all models were significantly lower than chance (χ^2^(1) > 3.84, *p* < 0.05) for rightward movements (Figure 7D). For movements into the response field, the error rate was significantly below chance only for variable rate model (χ^2^(1) = 4.00, *p* < 0.05).

We repeated the same analysis for decoding from LFP (Figures 7E–H). Again, mean ST was consistently lower for unified models (Figure 7F). Figure 7H shows the error rates for decoding each direction movement with the unified model, VSPN model and SPN model. For trials out of the response field, the error rate was lowest when the unified model was used. However, all three models performed well beyond chance level (χ^2^(1) > 3.84, *p* < 0.05; Figure 7H). For leftward movement decoding, only the SPN model yielded an error rate significantly below chance (χ^2^(1) = 6.43, *p* < 0.05), while the unified and VSPN models performed within chance level (χ^2^(1) < 3.84, *p* > 0.05).

Finally, we compute correlations between trial-by-trial STs from spiking and LFP data and SRT (Figures 7I–K) or RRT (Figures 7L–N). We also look at correlations between STs computed from spiking and LFP data (Figure 7O). Using the unified model, STs from LFPs for movements out of the response field were correlated significantly with SRT (*r* = 0.40, *p* < 0.001) and spike STs for movements into the response field were weakly correlated with RRT (*r* = 0.24, *p* = 0.05). The correlations for all other combinations of STs and reaction times were negligible. Correlations between spike-LFP STs were negligible for all models and movement directions. To accurately compare the effectiveness of different models from *p*-values of latency-behavior correlations, we needed to account for the multiple comparison problem—that by chance alone one of the models can yield significant correlation. The significance of only LFP discrimination latency—SRT correlation for rightwards movement using the unified model (Figure 7I) survived a Bonferroni correction (*N* = 24 models) at *p* < 0.05.

### Cued memory-guided saccades

We demonstrate the contribution of parametric models in the estimation of spike-LFP correlations by applying them to previously published data sets (Banerjee et al., 2010) in which simpler models (rate model for spikes and SPN model for LFPs) were used for decoding visual response onsets from spike-field recordings. Monkey B performed a cued memory guided saccade task in which a visual target flashed at a peripheral location directed a saccade to the remembered location. We used three example spike-field sessions for illustration of modeling and application of AccLLR procedure. The same examples were presented in Banerjee et al. (2010). In each session we computed how long after illumination of the visual stimulus the neural activity becomes selective for the visual stimulus (visual response onset).

**Figure 8:**
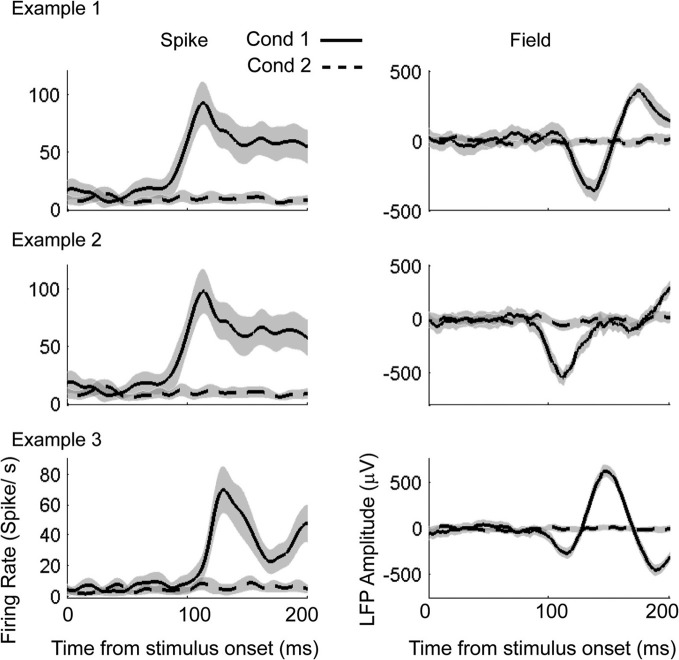
**Example spike-field recordings to detect visual response onset times. Left column**: Mean firing rate for condition 1 (solid) and condition 2 (dashed). Gray shading indicates the 95% confidence interval. **Right column**: Mean LFP for condition 1 (solid) and condition 2 (dashed).

To compute the visual response onset times using the AccLLR procedure, we needed to define two models from different data epochs. The choice of these two models defines the nature of the information processing that underlies the ST. We defined model 1 using activity recorded for 200 ms immediately following the onset of the visual stimulus (condition 1; Figure 8). We defined model 2 using activity during a 200 ms baseline period involving stable fixation but no peripheral visual stimulation (condition 2). We assessed the likelihood of the data belonging to each model category and accumulated the log-likelihood ratio in time until there was sufficient evidence in support of either model. If sufficient evidence was not obtained in support of either model within 200 ms, we declared the trial as “don't know.” We performed the AccLLR analysis on three example spike-field sessions (Figure 8) using the unified model, the variable signal model and the noise models. The results from the noise models were presented previously (Banerjee et al., 2010). Here, we study the performance of the variable signal model and the unified model as applied to the same spike-field sessions.

**Figure 9:**
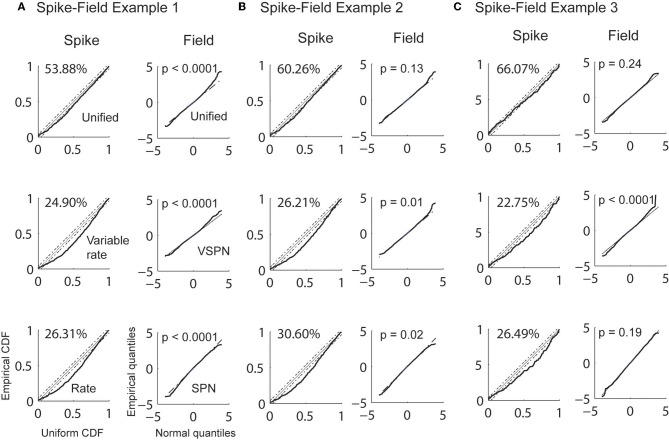
**Goodness of fit analysis for 3 spike-field sessions (A, B, and C).** Empirical CDFs of rescaled inter-spike intervals using conditional intensities function of unified model (1st row), variable signal (rate) model (2nd row), and noise (rate) model (3rd row) are plotted against uniform CDF for each spike-field example (1st, 3rd, and 5th column). The percentages of points that falls within 95% confidence intervals (CI) of a uniform theoretical CDF distribution are reported. Quantiles of LFP residuals from unified model (1st row), VSPN model (2nd row) and SPN model (3rd row) are plotted against standard normal quantiles for each spike-field example (2nd, 4th, and 6th column). *p*-values of K–S tests performed on the residuals are reported.

Estimation of visual response onset times is a detection problem as opposed to a discrimination problem for the cued look-and-reach task. Here, we estimated the history parameters, the GLM coefficients for the spikes and the AR coefficients for the LFP from the entire duration of recordings (200 ms) in the baseline condition (condition 2 in Figure 8). We then estimated the trial-by-trial amplitude scaling factors and latency lags in Condition 1 using the Bayesian estimation procedure. The goodness of fit was then tested for each model by CDF plots (Brown et al., 2002) for spikes. The empirical cumulative distribution of rescaled ISIs (rescaled by the time-rescaling theorem) are plotted against the cumulative distribution of a uniform distribution (Figure 9). The percentage of points captured by a model (goodness of fit) within 95% confidence level was always greatest for the unified model fits for each spike session. For LFPs, the goodness of fit of the residuals were computed using K–S tests statistics. For presentation, we plot quantiles of empirical distribution against the quantiles of a standard normal distribution. Here, the null hypothesis of the K–S tests is that the distribution of residuals is a standard normal distribution. Therefore, proximity of residual data to a standard normal distribution is expressed by the *p*-value. A significantly high *p*-value implies that the residual consists of only normally distributed data points from random noise and that the signal is sufficiently explained by the model. When the unified model was used, two out of three LFP sessions had *p* > 0.05 (null hypothesis true) indicating favorable model-to-data fit (Figure 9). When the SPN model was used, only session three indicated a favorable model-to-data fit. Residuals of the VSPN model fits always resulted in *p* < 0.05, indicating that the null hypothesis of normally distributed residuals may be rejected.

**Figure 10:**
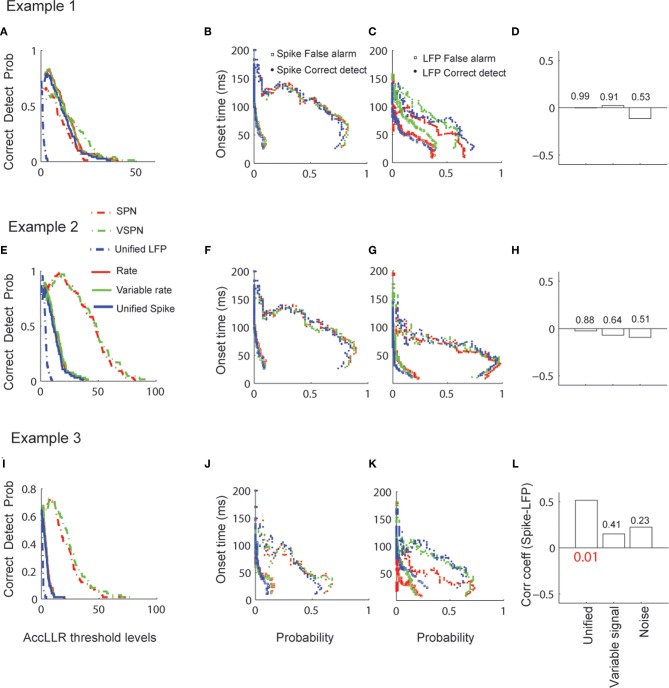
**Decoding of visual response onset times via AccLLR analysis. (A)** Correct detection probability (probability that an onset time is detected from Condition 1 trials of Figure 8, Example 1) as a function of AccLLR threshold levels plotted for unified model (blue), variable signal (green) and noise (red) models corresponding to spike trains (solid) and LFP (dashed). **(B)** Visual response onset times from spike trains (in spike-field example 1) versus probability curves with same color code as in **(A)**. Visual response onset times corresponding to false alarm probabilities (square) and correct detect probabilities (circles) are plotted. **(C)** Visual response onset times from LFP (in spike-field example 1) versus probability curves with same color code as in **(A)**. Visual response onset times corresponding to false alarm probabilities (square) and correct detect probabilities (circles) are plotted. **(D)** Correlation between visual response onset times from spikes and LFP. **(E–H)** Same as in **(A–D)** for spike-field example 2. **(I–L)** Same as in **(A–D)** for spike-field example 3. *P*-value that survives Bonferroni correction is reported in red font.

We applied the AccLLR analysis on all spike-field sessions and used the unified model, the variable signal model and noise models (Figures 10A–L). The correct detect probability (true positives) of decoding the visual response onset time from spike trains in Condition 1 did not show much variation across different models and within each session (Figures 10A,E,I) but varied across different threshold levels for AccLLR analysis. The probability of detecting visual response onset times using the SPN model or the VSPN model for LFP are similar. Overall, the correct detect probability versus AccLLR threshold level increased to a point and then decreased. Each threshold level generated empirical false alarm rates (from condition 2 trials) and correct detect probability rates (from condition 1 trials). We plot the mean of single trial visual response onset times obtained for different false alarm and correct detect probabilities (Figures 10B,C,F,G,J,K) to show the speed–accuracy trade off in detecting visual response onset times. An ideal threshold is one which maximizes the correct detect probability rates while minimizing the false alarm probability. A lower threshold results in faster visual response onset times but also higher false alarm rates. The general features of signal selectivity did not change across the spike train models (Figures 10B,F,J). However, for LFP, the mean visual response onset—probability curves showed variability across models within a session (Figures 10C,G,K). In sessions one and two, the false alarm rate was always lowest for the unified model compared to the VSPN model and the SPN model. In the third session, the false alarm rate for the unified model increased compared to the other two models as the AccLLR threshold was lowered to capture faster onset time. We computed the visual response onset times from spikes and LFPs by setting the AccLLR threshold to a point where the false alarm probability was less than the probability of detecting true negatives. All spike-field models (unified, variable signal and noise) resulted in error rates below chance level. For spikes, the unified model had the highest error rate in all sessions (mean 33% trials across sessions) compared to the variable rate model (mean 23% across sessions) and the rate model (mean 23% across sessions). For LFPs, error rates in the unified model (mean 30% trials across sessions) were lower than in the VSPN model (mean 33% across sessions) but higher than in the SPN model (mean 23% across sessions). Significant spike-LFP correlation in onset times (*r* = 0.5, *p* < 0.05, Bonferroni corrected) was observed for session three only while using the unified model (Figure 10L). None of the other sessions exhibited significant spike-LFP correlation in single trial visual response onset times (Figures 10D,H,L).

## Summary and discussion

We have proposed a family of statistical models to relate the processing of task-relevant variables with spike train and LFP dynamics. Many studies have shown that comparison between LFP and spike trains can be used to study task-specific neural circuits (Nielsen et al., 2006; Buschman and Miller, 2007; Monosov et al., 2008; Pesaran et al., 2008; Dean et al., 2012). However, to measure timing of neural responses, most of these studies compare the raw spike-field measurements with standard statistical tools (e.g., *t*-tests, ANOVA, etc.) which do not acknowledge the differences in statistical properties of these measurements.

In comparison, our framework decomposes the decoding of information processing into two steps. In the first step, a unified information-processing model is used to account for the data points in two alternate tasks. In the second step, estimation of the timing of information processing is decoded by transforming the time evolution of neural activity into the space of accumulated log-likelihood ratios based on the parametric models from step 1 (Banerjee et al., 2010). Accumulation of log-likelihood ratios to threshold has previously been shown to be an efficient way to decode the timing of information processing in single trials. The unified modeling framework that we have proposed is a natural extension of the existing modeling strategies: Poisson firing rate (Richmond and Optican, 1987) and variable rate (Bollimunta et al., 2007) models for spikes and SPN/VSPN (Truccolo et al., 2002) and AR process models for LFP (Ding et al., 2000). These previous models can be shown to be the limit case scenarios of the unified model for spiking and LFP respectively.

One key advantage of the unified modeling framework is that analogous parameters are used to model spike trains and LFP time series. Thus, even though spike trains and LFP waveforms have different statistical properties, a comparison between the trial-by-trial parameters can be performed. Such comparisons can be helpful for understanding information processing from spike trains and LFP recorded locally within an area or across brain areas.

The unified model is readily usable with other decoding frameworks (Gollisch and Meister, 2008; Butts et al., 2011; Schaub and Schultz, 2011). The main motivation behind this study was to better describe trial-by-trial variability present in a given spike-field data set with equivalent information processing parameters on a case-by-case basis. Next, we discuss the unified model in terms of three key features that underline its importance: (1) enhancement in goodness of fit of data points, (2) improvement in the performance of decoding information processing with AccLLR, and (3) ability to compare the timing of information processing between spikes and LFP signals.

### Enhancement of goodness of fit

Prior work on modeling of spike trains (Barbieri et al., 2001; Brown et al., 2002; Truccolo et al., 2005; Haslinger et al., 2010) and LFPs (Banerjee et al., 2010) to decode task-relevant events has argued that goodness of fit of models to experimental recordings is needed for model validation. We have validated unified spike-field models in simulated data where the independent variables (amplitude scaling factors/latency lags) are known, as well as in experimental recordings. The analysis on simulated data reveals that under varying noise to signal ratios, the unified model most accurately reconstructs the data (Figures 2 and 4). For data collected in experimental recordings, parameter estimation and model validation is performed in either different trials (for input shapes) or in different time windows (for ongoing background, GLM, and AR coefficients). We compared the performance of the unified model, variable signal models and noise models on experimental data through multiple reliability tests and ranked them accordingly in Table 1. Based on their performance in four spike-field sessions (one for cued look-and-reach task and three for memory guided saccade task), we have five data sets for evaluating overall modeling performance: left and right direction movement trials from a cued look-and-reach task and three memory guided saccade sessions. A reasonable performance in each scenario, e.g., goodness of fit at 95% confidence determined from K–S tests, significantly low error rates for detection of movement direction or response onset; is assigned a reliability score of unity. A winner takes all rule for scoring is applied for the AIC, false alarm rate and CDF plots such that only the model with best quantitative value gets a reliability score. Based on such a scoring rule, we conclude that the unified model has the best overall reliability and goodness of fit indices (Table 1).

**Table 1:** **Overall performance scores of models**.

**Measures**	**Unified model**	**Variable signal**	**Noise**
**SPIKES**			
		**Variable rate**	**Rate**
AIC	5	0	0
CDF plots	5	0	0
Error rates on single trials	5	3	3
False alarm rate	2	2	3
**FIELDS**			
		**VSPN**	**SPN**
AIC	5	0	0
K–S test on residual (*p* > 0.05)	2	0	1
Error rates on single trial	4	4	5
False alarm rate	1	3	1
**Total**	**29**	**12**	**13**

Spike-field models are scored according to various reliability measures in five sessions. A score of five means a model is acceptable in five sessions according to the corresponding reliability measure, four means in four sessions and so on. For AIC, False alarm rate and CDF plot, the model with lowest quantitative value takes all points. If there is a tie, a point is given to both models. An acceptable model is considered for K–S tests when p values > 0.05 and error rates for correct visual response onset/movement direction selection are less than chance level.

### Decoding task-relevant events on single trials

Decoding and information processing approaches have been identified as two ways to develop an understanding of trial-by-trial goal-directed behavior (Quian Quiroga and Panzeri, 2009). Multi-area recordings are ideally suited for studies that assess information processing at a network level (Cottaris and Elfar, 2009; Quian Quiroga and Panzeri, 2009; Salimpour et al., 2011). The unified model we present here provides a quantitative approach to extract information processing events in spike trains and LFP signals and relate them to cognitive components embedded in a task. For example, in the cued look-and-reach task, only the unified model could decode the movement direction well beyond chance level before the saccade initiation (SRT). Hence, we conclude that modeling background processes and trial-by-trial variability of input signals forms a minimum set of parameters that need to be tracked in spike-field recordings for studying action initiation at millisecond temporal resolution. Even though the unified models fared favorably in overall reliability rankings of Table 1, the variable signal and noise models were comparable when measures such as error rates and false alarm rates were considered. In fact, for LFPs the VSPN model seemed to have a better control of false alarm rates in more sessions (Table 1). Hence, a single statistical model may not be suitable for every recording session, but an overarching modeling framework that can be tuned for a study in hand is a more pragmatic choice. Future work with larger experimental data sets may help to address if accounting of information processing parameters reduces error rates, false alarm rates better than variable signal and noise models.

The unified model can be readily applied in likelihood-based decoding frameworks (Wiener and Richmond, 2002; Cottaris and Elfar, 2009; Quian Quiroga and Panzeri, 2009; Banerjee et al., 2010; Salimpour et al., 2011) to detect onsets of information processing stages during trial-by-trial goal-directed behavior. The information processing parameters can be used along with the response onset times to address the relationship of neural events with measures of behavioral performance such as reaction times or decision times (DiCarlo and Maunsell, 2005). Simultaneously measuring neural latencies from different brain areas may reveal the functional organization of the brain at the network level. The techniques developed for decoding information processing in LFPs can also be extended to electroencephalogram (EEG), magnetoencephalogram (MEG) and intra-cranial EEG recordings in humans without difficulty.

### Comparison across information processing in spikes and field

Correlation in the spectral domain between spikes and fields within an area or across multiple areas is an effective way to understand the neuronal mechanisms of goal-directed behavior (Pesaran et al., 2002; Brown et al., 2004; Miller and Wilson, 2008; Pesaran et al., 2008; Quian Quiroga and Panzeri, 2009). In recent work, we have presented a statistical framework that permits a direct comparison of spike and LFP ST calculations (Banerjee et al., 2010). The analysis of spike-field correlations presented here extends that work and suggests that the unified model may be useful in extracting such information at the single trial level.

Using the unified model, we find that significant correlations (*p* < 0.05 Bonferroni corrected) exist between the SRT and STs computed from LFPs in area LIP before cued look-and-reach movements into the response field of the site under study. We do not observe correlations between behavior and spike/LFP STs or between STs from spikes and LFP using other modeling strategies (variable signal and noise). In a second task using memory guided saccades, we observe a strong correlation between visual response onsets computed using spike trains or LFP in one of three sessions (example 3) only when the unified model is used (*r* = 0.5, *p* < 0.05, Bonferroni corrected). This result corresponds with our earlier results of spike-field onset time correlations observed from trial averaged accumulated log-likelihood ratios (Banerjee et al., 2010). These preliminary results suggest that the unified model can be used to explore spike-field onset time correlations in the future.

Employing GLM and AR parameters in the unified modeling framework may permit extensions of other measures such as Granger causality (Chen et al., 2006; Nedungadi et al., 2009; Kim et al., 2011) in spike-spike, field-field, and spike-field recordings. Based on the goodness of fit obtained from unified models of spike-field time series, one can develop comparable measures of variance from spike-field data set. This will be an important step in future research and extensions of the unified modeling framework.

### Conflict of interest statement

The authors declare that the research was conducted in the absence of any commercial or financial relationships that could be construed as a potential conflict of interest.
